# Histological and immunological differences between zoonotic cutaneous leishmaniasis due to *Leishmania major* and sporadic cutaneous leishmaniasis due to *Leishmania infantum*

**DOI:** 10.1051/parasite/2019007

**Published:** 2019-02-27

**Authors:** Thouraya Boussoffara, Mohamed Samir Boubaker, Melika Ben Ahmed, Mourad Mokni, Ikram Guizani, Afif Ben Salah, Hechmi Louzir

**Affiliations:** 1 Laboratory of Transmission, Control, and Immunobiology of Infections, Pasteur Institute of Tunis Tunis Tunisia; 2 Laboratory of Human and Experimental Pathology, Pasteur Institute of Tunis Tunis Tunisia; 3 Laboratory of Molecular Epidemiology and Experimental Pathology Applied to Infectious Diseases, Pasteur Institute of Tunis Tunis Tunisia; 4 University of Tunis El Manar Tunis Tunisia; 5 Department of Dermatology, Hospital La Rabta Tunis Tunisia; 6 Faculté de Médecine de Tunis Tunis Tunisia; 7 Department of Family and Community Medicine, College of Medicine and Medical Sciences, Arabian Gulf University (AGU) Manama Bahrain

**Keywords:** *Leishmania major*, *Leishmania infantum*, lesion, cytokines, chemokines

## Abstract

Lesion features in cutaneous leishmaniasis (CL) depend on the infecting *Leishmania* species as well as on host immune reponse. In this study, we evaluated the histological and immunological differences between two forms of CL described in Tunisia: zoonotic cutaneous leishmaniasis (ZCL) caused by *L. major* and sporadic cutaneous leishmaniasis (SCL) caused by *L. infantum*. Histological analysis showed a mild to moderate infiltrate within ZCL lesions. In contrast, massive infiltration of the dermis was observed within SCL lesions. Contrary to ZCL, infiltrates within SCL lesions were organized and showed granuloma composed of macrophages and lymphocytes. In addition, immunohistochemical analysis showed a predominance of CD4^+^ T cells within both CL forms. Furthermore, expression of interferon-γ, interleukin (IL)-10, IL-8, IL-13 and monocyte chemotactic protein (MCP)-1 was evaluated using real-time quantitative polymerase chain reaction (RT-qPCR). MCP-1 and IL-10 were expressed at comparable levels in ZCL and SCL lesions. Interestingly, IL-8 mRNA levels were significantly higher in ZCL lesions compared to SCL lesions, but interferon-γ was significantly higher in SCL lesions than in ZCL lesions.

## Abstract (fr)

Les caractéristiques des lésions de la leishmaniose cutanée (LC) dépendent de l’espèce de *Leishmania* infectante ainsi que de la réponse immunitaire de l’hôte. Dans cette étude, nous avons évalué les différences histologiques et immunologiques entre deux formes de LC décrites en Tunisie, la leishmaniose cutanée zoonotique (LCZ) causée par *L. major* et la leishmaniose cutanée sporadique (LCS) causée par *L. infantum*. L’analyse histologique a montré un infiltrat léger à modéré dans les lésions de LCZ. En revanche, une infiltration massive du derme a été observée dans les lésions de LCS. Contrairement à la LCZ, les infiltrats au sein des lésions LCS étaient organisés et montraient un granulome composé de macrophages et de lymphocytes. En outre, l’analyse immunohistochimique a montré une prédominance de cellules T CD4^+^ dans les deux formes de LC. De plus, l’expression de l’interféron-γ, des interleukines (IL)-10, IL-8, IL-13 et de la protéine chimiotactique monocytaire (MCP)-1 ont été évaluées en utilisant une réaction en chaîne de la polymérase quantitative en temps réel (RT-qPCR). MCP-1 et IL-10 étaient exprimés à des niveaux comparables dans les lésions LCZ et LCS. Fait intéressant, les niveaux d’ARNm d’IL-8 étaient significativement plus élevés dans les lésions LCZ que dans les lésions LCS, mais l’interféron-γ était significativement plus élevé dans les lésions LCS que dans les lésions LCZ.

## Introduction

In North Africa, cutaneous leishmaniasis (CL) is caused by three *Leishmania* (*L.*) species: *L. major*, *L. tropica*, and *L. infantum*. These three species prevail under different bioclimates and differ by the nature of their vectors and reservoir hosts [4]. They are responsible for three CL forms, which exhibit different epidemiological and clinical features, and need specific control measures. In Tunisia, the most prevalent CL form is zoonotic cutaneous leishmaniasis (ZCL), caused by *L. major*. ZCL is endemic in several rural areas of the southern and central regions of Tunisia [39]. Transmission of *L. major* by the phlebotomine sand fly vector, *P. papatasi*, occurs during summer and active lesions in humans emerge during the autumn and winter months [6]. The disease is pleomorphic in its clinical presentation and course. In North Tunisia, sporadic cutaneous leishmaniasis (SCL), caused by *L. infantum*, is described [3]. Its distribution overlaps with visceral leishmaniasis, with an incidence of approximatly 30 cases per year [21]. SCL is caracterized by single ulcerated dermal lesions that localize mostly in the face. The third form is chronic cutaneous leishmaniasis (CCL) caused by *L. killicki*, an enzymatic variant of *L. tropica* which occurs within microfoci in south-eastern Tunisia [7]. New foci recently emerged in the central and south-western parts of the country [10].

The outcome of *Leishmania* infection depends on the parasite, host and sandfly [34]. It depends on a complex set of interactions between several factors triggered by host innate and acquired immune responses [40]. In fact, the most important line of defense against parasite infection is cell-mediated immune response, which participates actively in granuloma formation that will eventually limit the expansion of the infectious agent and thus control the disease [25]. Accordingly, several studies have focused on intralesional cytokine and chemokine gene expression, especially in human New World leishmaniasis [20, 26, 33, 43], but few studies have been conducted concerning Old World cutaneous leishmaniasis [23]. During *L. braziliensis* infection, Th1 cytokine mRNAs (IFN-γ and lymphotoxin) are present in localized cutaneous lesions, whereas IL-4, IL-5, and IL-10 mRNAs are abundant in mucosal lesions [33]. Similarly, mRNAs of IFN-γ, TNF-α, and IL-8 were found to be expressed in all forms of American cutaneous leishmaniasis (ACL), whereas IL-4, IL-5, and IL-10 were expressed in mucosal and diffuse forms of the disease [12]. Th2 cytokines were weakly expressed in localized cutaneous leishmaniasis (LCL) caused by *L. braziliensis* or *L. major* compared to Th1 cytokines [12, 23, 26, 33]. However, we showed in a previous study that the presence of Th1 cytokines (IL-12 and IFNγ) is not correlated with the healing process of the ZCL lesions due to *L. major* infection. High levels of these protective cytokines were detected in lesions with a protracted clinical course compared to those showing clinical improvement, indicating that the pathogenesis of the disease is not related to inadequate Th1 cell response [23]. In addition, in localized ACL caused by *L. mexicana,* intralesional expression of IL-2, IL-3, IL-4, and IL-5 was minimal or absent, whereas IL-1α, IL-6, IL-10, TGF-β, IFN-γ, and TNF-α mRNAs were abundant [26]. However, it was shown that in cutaneous leishmaniasis due to *L. guyanensis*, Th2 cytokines, and particularly IL-13 are produced locally at the site of infection [8]. Authors demonstrated that Th2 response precedes the development of a Th1 response at the local site of infection in LCL patients [9].

In the present study, we first evaluated whether the two forms of LCL (ZCL and SCL) showed any differences in histopathological and immunohistochemical features. Then, using an RT-qPCR method, we compared the intra-lesional expression of certain cytokines (IFN-γ, IL-10 and IL-13) and chemokines (IL-8 and monocyte chemotactic protein (MCP)-1) in an attempt to evaluate immune reponse within lesions.

## Materials and methods

### Ethical issues

Skin lesion specimens examined in this study were collected between 1996 and 2000 within the framework of several projects conducted at the Pasteur Institute of Tunis. The protocols were approved by the Bio-Medical Ethics Committee (BMEC) of the Pasteur Institute of Tunis.

### Skin lesion specimens

Skin lesion specimens were obtained from two groups of patients. The first was composed of 20 patients with active ZCL (age range 7–14 years, mean age 9.9 years with a sex ratio equal to 0.66) living in an endemic focus of *L. major* transmission (governorate of Kairouan in the center of Tunisia). Patients with multiple lesions had two to three biopsies done. The second group was composed of 32 patients with active SCL (age range 10–76 years, mean age 26.5 with a sex ratio equal to 1.28) living in an endemic focus of *L. infantum* transmission (governorate of Beja in northern Tunisia). Serous dermal fluid was first collected from the border of the lesion and was used in parasitological tests, as described below. Thereafter, a 3 mm diameter skin punch biopsy was taken from each patient after local anesthesia at the border of the lesion. Lesion specimens were divided into two parts: one was immediately snap-frozen in liquid nitrogen, and the other was fixed in 10% neutral formalin saline for paraffin embedding. As negative controls, skin biopsies were obtained from eight donors suffering from other diseases (age range 27–59 years, mean age 39.4 years) with a sex ratio equal to 0.66.

### Parasitological evaluation

For the detection of *Leishmania* parasite, we used the protocol previously described [23]. Briefly, serous dermal fluid was collected from the border of the lesion and used to prepare May-Grünwald-Giemsa (MGG)-stained smears and to inoculate Novy-MacNeal-Nicolle (NNN) medium or coagulated rabbit serum (CRS) medium. The intralesional parasite burden was estimated by counting amastigotes on MGG-stained smears. SCL lesions were selected on the basis of the positivity of one of these tests. Otherwise, the *Leishmania* parasites that had been isolated in NNN or CRS medium were then expanded in RPMI 1640 medium containing 10% heat-inactivated fetal calf serum and used for the purpose of typing strains either by isoenzyme electrophoresis or molecular techniques [5, 21]. Species identification was carried out by our collaborators in the framework of other projects aiming to characterize the geographical distribution of *Leishmania* species in Tunisia [3, 5]. The main *Leishmania* species isolated from SCL lesions is *L. infantum* zymodemes MON-24 and that isolated from ZCL lesions is *L. major* MON-25 [21].

### Histopathological analysis

Tissue sections 3–4 μm thick were cut from formalin fixed, paraffin-embedded blocks and were used to prepare routine histopathology and immunohistochemistry (IHC) slides. Inflammatory reactions in samples were based on the infiltrate density, presence or absence of plasma cells, macrophages (epithelioid cells and giant cells), unorganized or organized granulomas, and type of epithelial hyperplasia in stained slides. Cells were assessed using a semi-quantitative procedure (slight to intense). Evaluation of the lymphocytic infiltrate, plasmocytes and polynuclear density was performed as previously described by Mokni et al. [28], according to the following six-stage classification: absent (0), slight (+/−), moderate (+), slight intense (++), intense (+++), and very intense (++++). The type of epithelioid granuloma was evaluated according to the following three-stage classification: 0 = absent, 1 = disorganized granuloma, and 2 = organized granuloma. Epithelial hyperplasia was evaluated according to the following four-stage classification: 0 = none, 1 = slight, 2 = moderate, and 3 = pseudo EOA (pseudoepitheliomatous hyperplasia).

### Immunohistochemical study

Immunohistochemical staining was performed by the labeled streptavidin biotin visualization system (LSAB) using an LSAB-HRP kit (DakoCytomation, Denmark A/S), according to the manufacturer’s instructions. The following primary monoclonal antibodies were used for immunohistochemical staining: rabbit anti-human CD3 and mouse anti-human CD4, CD8, CD56 from Becton Dickinson (San Jose, California). Briefly, 4 μm thick cryostat sections were air dried for at least 2 h, then fixed in acetone for 3–5 min, dried and washed twice in phosphate-buffered saline (PBS; pH 7.2) for 5 min. Slides were then incubated with blocking solution for 5 min before adding primary antibodies at the appropriate dilution for 30 min in a humid atmosphere at room temperature. The sections were washed in PBS and then allowed to react for 10 min with biotinylated anti-mouse or anti-rabbit antibodies (DakoCytomation, Denmark A/S). The slides were rinsed in PBS and subsequently incubated with streptavidin-HRP (DakoCytomation, Denmark A/S) for the next 25 min. After a 10 min rinse in PBS, slides were developed using the AEC substrate system and counterstained with 1% hematoxylin. Slides were analyzed under a light microscope at a magnification ×400 by two independent investigators in a blinded fashion. For all antibodies, positively stained mononuclear cells were counted by observing ten randomly chosen fields for each sample. The results are expressed as percentage of positively stained cells among mononuclear cells.

### Quantification of gene expression by quantitative real-time PCR

Total RNA was extracted from frozen lesion specimens using the TRIzol^®^ reagent (Gibco BRL. Life Technologies, Inc.), according to the manufacturer’s instructions. cDNA was synthesized from 1 μg of total RNA using reverse transcriptase M-MLV RT (Murine-Moloney Leukemia virus reverse transcriptase, Gibco BRL, Gaithersburg, MD). mRNA quantification was performed for IFN-γ, IL-10, IL-13, IL-8 and MCP-1 using quantitative PCR (q-PCR) according to the TaqMan procedure and using the “Universal PCR Master Mix” and “Pre-developed assay reagent Kit” (Applied Biosystems, Foster City, CA). For each PCR reaction, target samples were tested with three negative controls. Following activation of the AmpliTaq Gold for 10 min at 95 °C, 40 cycles of denaturation at 95 °C for 15 s and annealing and extension at 62 °C for 1 min were carried out. Quantity of mRNA was firstly normalized referring to expression of an endogenous control, the 18S ribosomal RNA (18S rRNA), given by the formula 2^–∆CT^ (∆C_T_ is the difference in threshold cycles for target and reference), and then relative to a calibrator (Mean ∆C_T_ of each gene mRNA expression within biopsies from normal skin) given by the formula 2^–ΔΔCT^ (ΔΔC_T_ is the difference in ΔC_T_ for target and normal skin).

### Quantitative real-time PCR assay for parasite load

Parasite load within CL lesion specimens was evaluated by quantification of the KMP-11 (Kinetoplastid Membrane Protein) gene encoding for a protein abundantly expressed in promastigotes [22]. The PCR reaction was performed using a Taq-Man PCR kit (Applied Biosystems). The upstream and downstream primer sequences of KMP-11 were 5′-CGCCAAGTTCTTTGCGGACAA-3′ and 5′-CATGATCAGGGAGCACACA-3′, respectively. A fluorogenic probe 5′ FAM–CGCCCGAGATGAAGGAGCACTACG–TAMRA3′ was synthesized by PE Applied Biosystems (Foster City, CA). Each q-PCR reaction was set in a total of 25 μL each, containing 50 ng of template gDNA, 0.3 μM of each forward and reverse primer, 0.1 μM fluorogenic probe, 12.5 μL of q-PCR master mix and 5.75 μL nuclease-free water. In addition, a “no template” control in duplicate was included on each plate to prove absence of contamination. PCR conditions were as follows: initial denaturation at 95 °C for 10 min and 40 cycles at 95 °C for 15 s and 60 °C for 60 s. Parasite number within LC lesion specimens was determined using a standard curve developed by serial dilutions of *Leishmania* genomic DNA (4 × 10^9^ *L. major* promastigotes). All samples with *C*_T_ > 37 were considered negative and a minimum of two copies of the KMP-11 gene was detected. *C*_T_ values from skin specimens were plotted on the standard curve and the quantity of DNA was calculated. To determine the parasite number within CL lesion specimens, we assumed that the amount of DNA per *Leishmania* parasite equals 0.1 pg [29]. To calculate cell numbers in analyzed samples, we assumed that the amount of DNA per human cell equals 6 pg [38]. Results were expressed in number of parasites per 10^5^ human cells.

### Statistical analysis

Data analysis was performed using statistical software Graph-Pad Prism 5.03 for Windows. Nonparametric statistical tests were used. Comparison between ZCL and SCL lesions was done using a Mann–Whitney test and differences were considered statistically significant when *p* < 0.05. Correlations between continuous variables were evaluated using the Spearman’s rank correlation test.

## Results

### Clinical, histopathological and phenotypic characterization of lesions

The age of ZCL lesions ranged from 10 to 360 days (mean ± *SD*, 84.28 ± 84.28 days) with a surface area ranging from 78.5 to 1963.5 mm^2^ (mean ± *SD*, 677.9 ± 545.7 mm^2^). The lesions were ulcerated and crusted in 56.5% and nodulo-ulcerative in 43.5%. In contrast, all SCL patients presented a single lesion with a surface area ranging from 19.6 to 7853.9 mm^2^ (mean ± *SD*, 816.25 ± 1670.6 mm^2^). Lesion age ranged from 60 to 1080 days (mean ± *SD*, 279.28 ± 263.86 days) and the majority (21/30) were nodulo-ulcerative.

The semi-quantitative results of the histopathological study of ZCL and SCL lesions are summarized in Table 1. A significant statistical difference was found between the aspects of the dermal infiltrate within ZCL and SCL lesions (*p* < 0.0001). While massive infiltration of lymphocytes (+++, ++++) was observed within most (96%) SCL skin lesions, the majority of ZCL lesions (87%) showed moderate infiltration of the dermis (+/−, +) (*p* < 0.0001). In SCL lesions, the cell infiltrate contains few plasmocytes contrary to ZCL lesions but the difference was not statistically significant (*p* = 0.37). Interestingly, in SCL lesions, the infiltrates consisted of granuloma composed of macrophages and lymphocytes. Except for five SCL lesions, in which the granuloma was disorganized, the other lesions showed an organized granuloma. Foci of epithelioid and Langerhans’ giant cells were seen, suggesting the end of the process of intracellular parasite destruction. Interestingly, 68.75% of ZCL lesions showed a moderate to intense infiltrate of polynuclear cells (+ to +++). In contrast, these cells were absent or moderate within SCL lesions (0 to +). Plasma cells were often seen in dense clusters at the periphery of the granuloma. In contrast to ZCL, which showed slight to moderate epithelial hyperplasia, no epithelial hyperplasia or slight hyperplasia was observed in SCL lesions, and the difference between the two forms of leishmaniasis was statistically significant (*p* = 0.0175).

**Table 1:** Semi-quantitative results of the histological study of ZCL and SCL lesions.

Histological characteristics	Grading score	ZCL lesions (*n* = 16)	SCL lesions (*n* = 29)
Granuloma	Absent	15	1
	Disorganized	1	5
	Organized	0	23
Density of cells in the infiltrate			
Lymphocytes	0	0	0
	+/−	9	0
	+	5	0
	++	1	1
	+++	1	10
	++++	0	18
Plasmocytes	0	3	11
	+/−	2	5
	+	5	8
	++	4	5
	+++	2	0
	++++	0	0
Polynuclears	0	4	17
	+/−	4	10
	+	6	2
	++	1	0
	+++	1	0
	++++	0	0
Epithelial hyperplasia	Absent	1	18
	Weak	10	6
	Moderated	5	3
	Pseudo EAO	0	2

*n*: number of lesion specimens studied.

**Figure 1:**
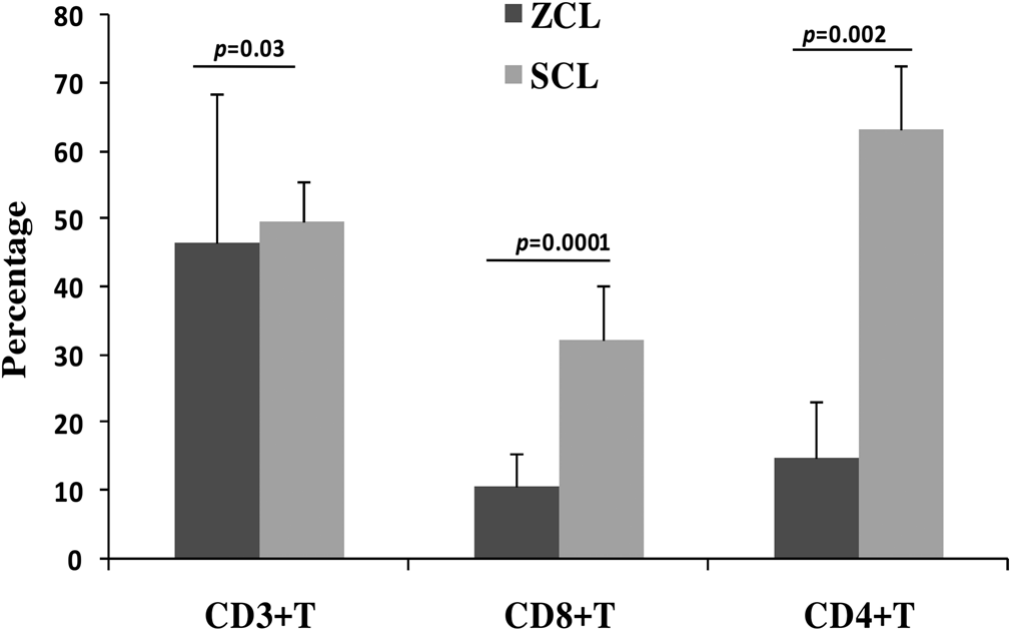
Proportions of CD3^+^, CD4^+^ and CD8^+^ T cells within ZCL and SCL lesions. Histograms represent results of the immunohistochemical analysis expressed as a percentage of positively stained cells among mononuclear cells. Results are expressed as the mean plus the standard error of percentage mean. Any significance found is indicated by a bar on top of each graph connecting the two comparisons. *p* < 0.05 was considered statistically significant.

The immunohistochemical study showed that the percentage of CD3^+^ T cells among the lymphocytic infiltrate varied from 25% to 97% (46.4 ± 21.8%) and from 38.8% to 59.7% (49.7 ± 5.85%) within ZCL and SCL lesions, respectively (Fig. 1). The percentage of CD8^+^ T cells among the mononuclear cells that compose the inflammatory infiltrates was significantly higher in SCL lesions (18.3%–45.5% (32.3% ± 7.9%) compared to ZCL lesions (5%–19.3% (mean ± *SD*, 10.7% ± 5.62%)) (*p* = 0.0001) (Fig. 1). Similar results were obtained with CD4^+^ T cells (Fig. 1). The percentage of CD4^+^ T cells was significantly higher in SCL lesions (56%–77% (63.2% ± 9.3%) compared to ZCL lesions (7%–27% (mean ± *SD*, 14.9% ± 8.4%)) (*p* = 0.002). A predominance of CD4^+^ T cells over CD8^+^ T cells, with a mean value for CD4^+^/CD8^+^ ratio of 1.4 and 1.2, respectively, was observed within lesions of ZCL and SCL. NK cells were not detected within ZCL or SCL lesions.

### Parasite load quantification within ZCL and SCL lesions

**Figure 2:**
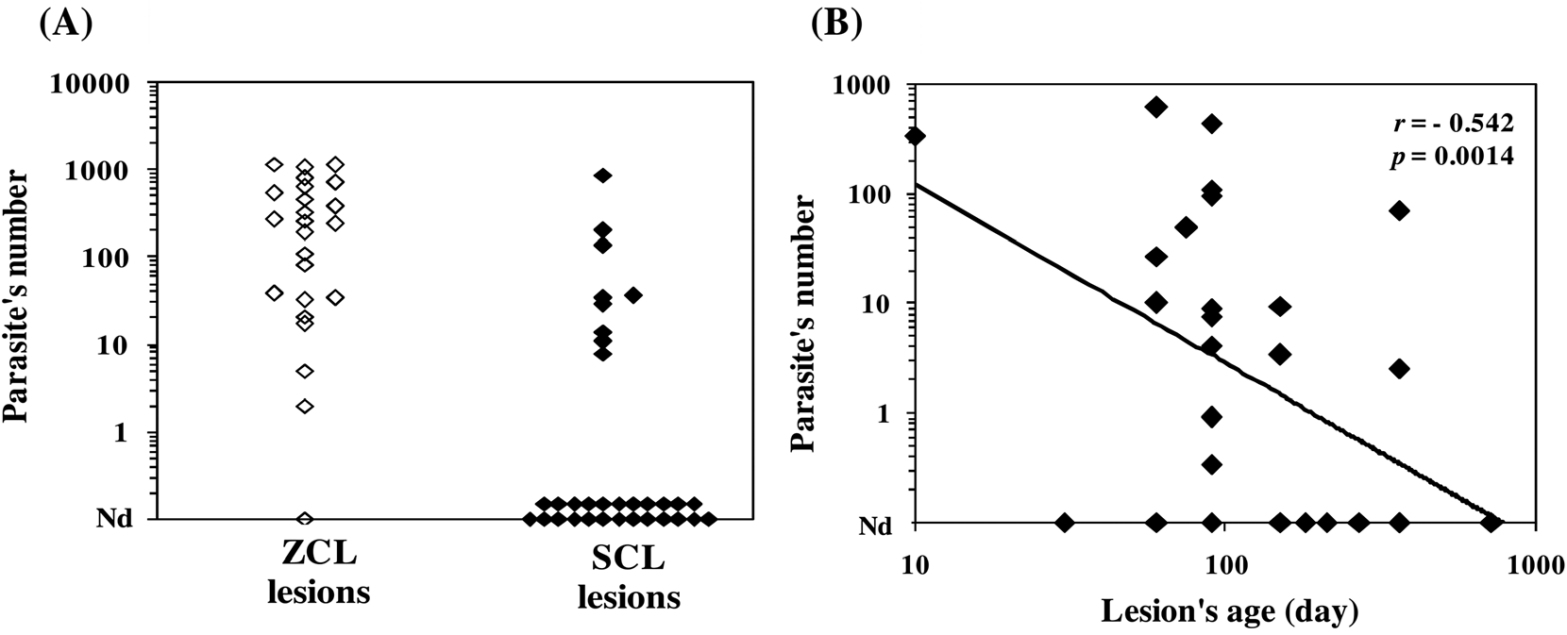
Parasite load within ZCL and SCL lesions. (A) Results were expressed as parasite number per 10^5^ human cells. (B) Expression of the number of parasites according to the age of the lesion. Correlation between lesion age and parasite number was evaluated using the Spearman’s rank correlation test, *r:* correlation coefficient; *p* < 0.05 was considered statistically significant. Nd; not detected.

The number of parasites was significantly higher in ZCL lesions compared to SCL lesions (mean: 151.1 ± 197.4 for ZCL vs. 21.6 ± 87.5 for SCL, *p* = 0.0001) (Fig. 2A). Accordingly, the *Leishmania* parasite was detected in 26/27 ZCL lesions with a wide range (2–1162 parasites/10^5^ human cells). By contrast, *Leishmania* was detected in only 9/32 SCL lesions (the parasite number varied from 8 to 200 parasites/10^5^ human cells) except for one lesion, which contained 879 parasites/10^5^ human cells. A negative correlation was found between the number of parasites and the age of the lesion (Spearman rank correlation *r* = −0.542; *p* = 0.0014) (Fig. 2B).

Results obtained using q-PCR were concordant at 72.7% and 58.8% with those obtained by inoculation of serous dermal fluid, collected from the border of the lesion, on NNN or CRS medium, respectively, and at 80% with those of May-Grünwald-Giemsa (MGG)-stained smears.

### Dominance of IFN-γ expression within CL lesions

**Figure 3:**
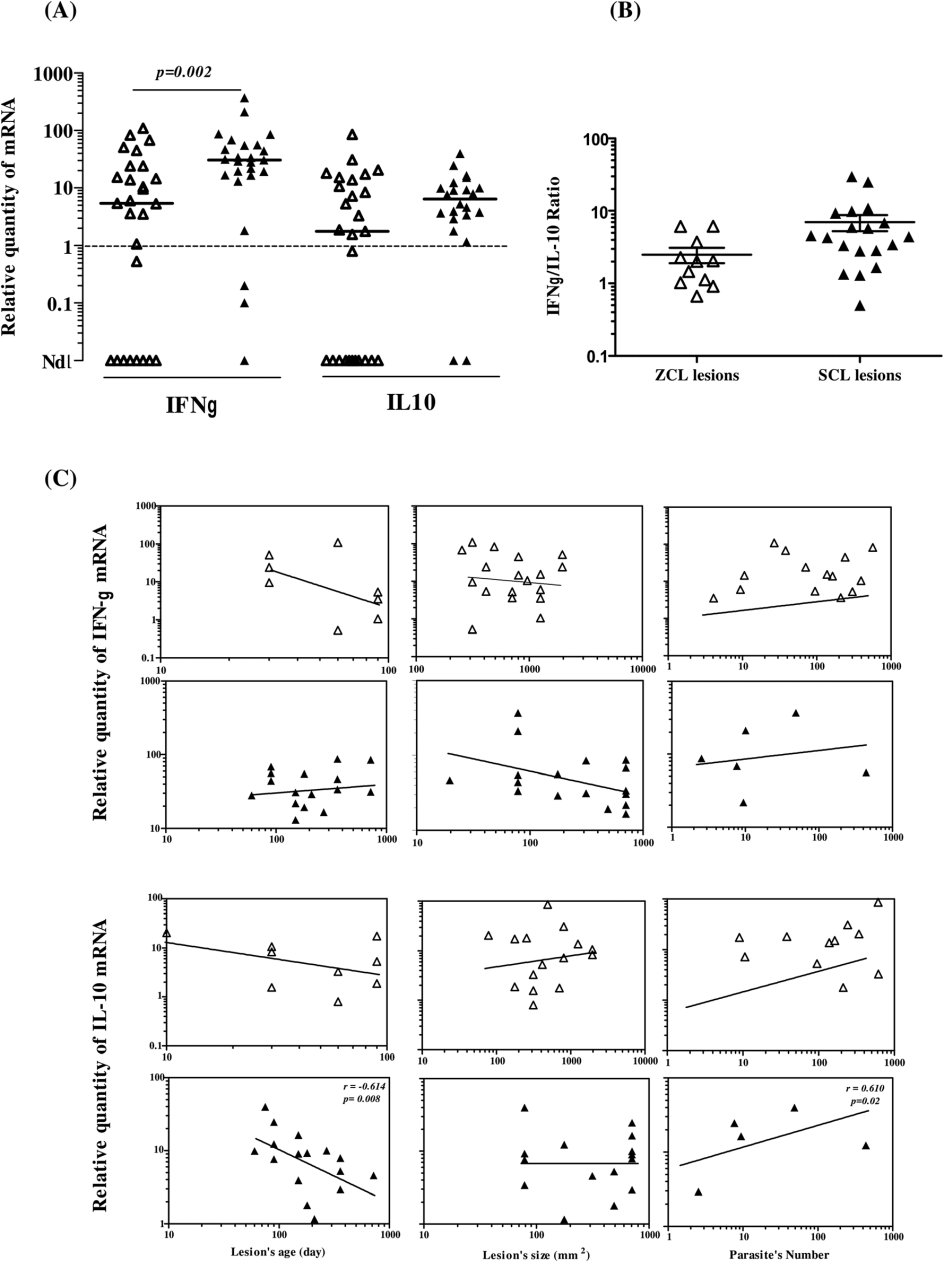
IFN-γ and IL-10 mRNA expression within ZCL and SCL lesions. (A) Dot-plots show individual values of IFN-γ or IL-10 mRNA expression within ZCL (Δ) and SCL (▲) lesions. A horizontal bar indicates the median for each group. Results are expressed as the ratio between levels of cytokine mRNA in samples studied to those detected in normal skin. Differences between groups were considered statistically significant when *p* < 0.05. Nd: not detected. (B) Dot-plots show the ratio of IFN-γ/IL-10 mRNA levels calculated for each lesion. Horizontal bars indicate mean and the standard error of mean (SEM) for each group. (C) Correlation between IFN-γ or IL-10 mRNA levels and the age of lesions, their size, and the number of parasites they contain. Correlations between different variables were evaluated with the Spearman’s rank correlation test, *r:* correlation coefficient; *p* < 0.05 was considered statistically significant.

To ascertain whether the pattern of cytokines in lesions could reflect the clinical course of CL, we examined IFN-γ, IL-10 and IL-13 expression in CL lesions by q-PCR. IL-13 mRNA was detected at low levels within only six ZCL and three SCL lesions (data not shown). In contrast, IFN-γ and IL-10 mRNAs were expressed at high levels within 77.27% (17/22) and 63.6% (14/22) of ZCL lesions, respectively (Fig. 3A). Similarly, these cytokines were detected within 86.36% (19/22) and 95.45% (21/22) of SCL lesions (Fig. 3A). Interestingly, IFN-γ mRNA expression was significantly higher in SCL compared to ZCL lesions (*p* = 0.002); however, no significant difference was observed for IL-10 mRNA expression. The ratio of IFN-γ to IL-10 mRNA levels indicated a predominance of IFN-γ (ratio > 1) in most of the biopsy samples, suggesting occurrence of a Th1 response (Fig. 3B). In SCL samples, the IL-10 mRNA levels correlate positively (*r* = 0.6108, *p* = 0.008) with the number of parasites, and negatively with the age of the lesion (*r* = −0.610, *p* = 0.02) (Fig. 3C).

### ZCL lesions express higher levels of IL-8 compared to SCL lesions

**Figure 4:**
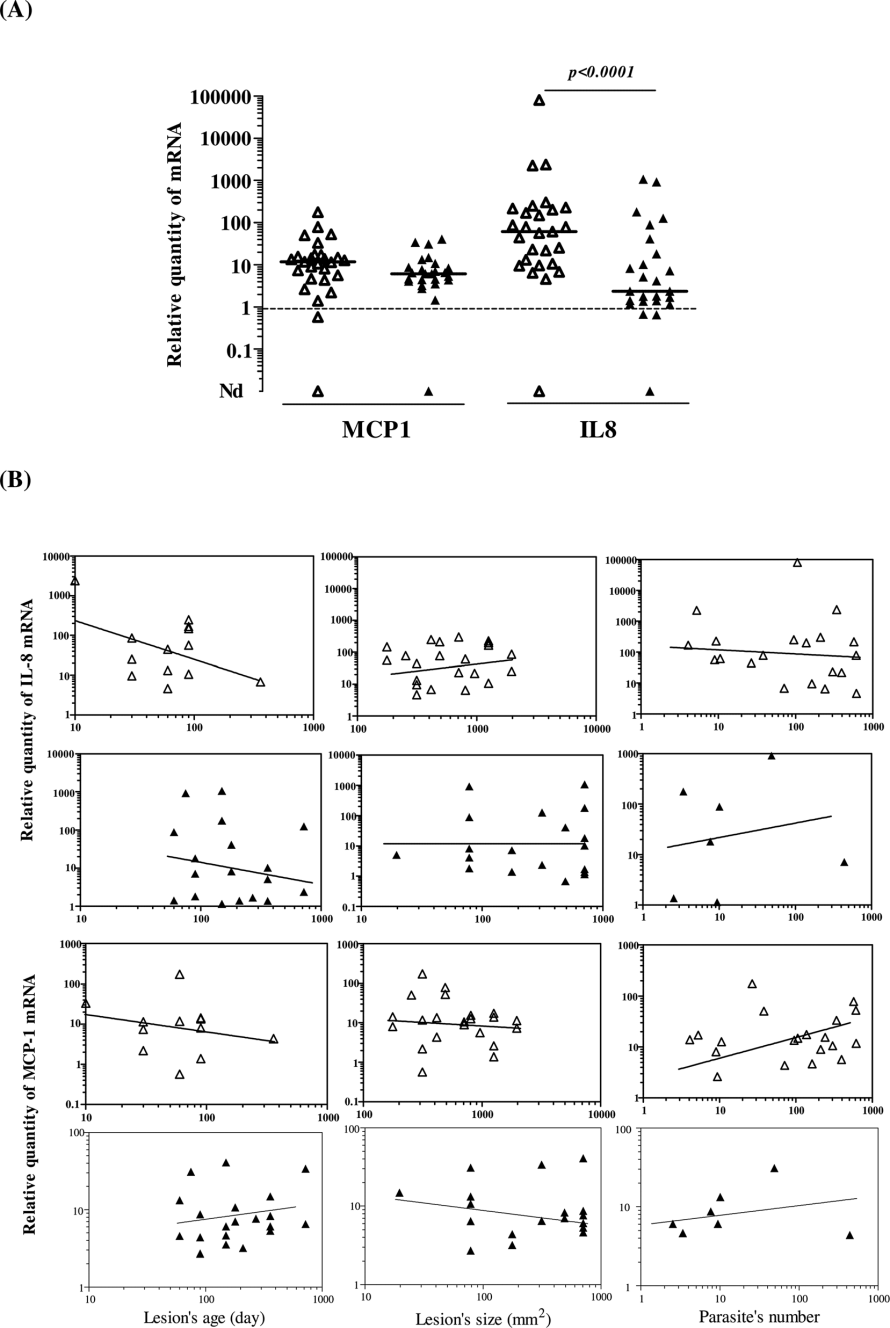
IL-8 and MCP-1 mRNA expression within ZCL and SCL lesions. (A) Dot-plots show individual values of IL-8 and MCP-1 mRNA expression within ZCL (Δ) and SCL (▲) samples. Results are expressed as a ratio between levels of mRNA molecules in studied samples to those detected in normal skin. A horizontal bar indicates the median value of each group. (B) Correlation between IL-8 or MCP-1 mRNA levels and the age of lesions, their size, and the number of parasites they contain. Correlations between different variables were evaluated with the Spearman’s rank correlation test, *r:* correlation coefficient; *p* < 0.05 was considered statistically significant.

Expression of pro-inflammatory chemokines, interleukin-8 (IL-8) and monocyte chemotactic factor-1 (MCP-1) was evaluated within the studied lesions. Almost all lesions expressed IL-8 mRNA and MCP-1 mRNA (Fig. 4). MCP-1 was expressed at comparable levels within ZCL and SCL lesions (*p* > 0.05). In contrast, IL-8 mRNA levels were significantly higher within ZCL lesions as compared to SCL lesions (*p* = 0.002). Interestingly, a positive correlation was found between levels of MCP-1 mRNA and those of IL-8 within either ZCL or SCL lesions (Table 2). However, no correlation was found between the levels of these chemokines and the lesions’ size, their age, or the number of parasites they contain.

**Table 2:** Correlation between mRNA expression levels of studied cytokines and chemokines within ZCL and SCL lesions.

		IL-10	IL-8	MCP-1
IFN-γ	ZCL	*r = 0.791*	*r* = −0.034	*r = 0.633*
		*p = 0.005*	*p = 0.889*	*p = 0.004*
	SCL	*r* = 0.291	*r = 0.590*	*r = 0.528*
		*p* = 0.226	*p = 0.004*	*p = 0.012*
IL-10	ZCL		*r* = 0.040	*r = 0.635*
			*p* = 0.884	*p = 0.008*
	SCL		*r* = 0.302	*r* = 0.381
			*p* = 0.196	*p* = 0.097
IL-8	ZCL			***r = 0.471***
				***p = 0.015***
	SCL			***r = 0.602***
				***p = 0.002***

*Note*. A Spearman rank correlation coefficient near 1 with *p* < 0.05 reflects a linear relationship in expression of molecules. IL, interleukin; IFN, interferon; MCP, monocyte chemotactic protein.

## Discussion

In cutaneous leishmaniasis, the severity of the disease is dependent on the parasite species and on the immune responses developed by the host [31]. Herein we analyzed the immune response within lesion of ZCL and SCL, two different forms of human CL described in Tunisia, caused respectively by *L. major* and *L. infantum* in an attempt to explore its involvement in the variability of clinical manifestations of the infection.

Histopathological analysis showed differences in the dermal infiltrate between both forms of CL. The SCL lesions were characterized by a massive dermal infiltrate with organized granuloma composed of macrophages and lymphocytes. By contrast, the ZCL lesions showed a high density of polynuclear cells recruited to the site of infection. The latter cells can be infected by the parasite, thus playing an important role in shaping the immune response against infection. Accordingly, as discussed by Scott and Novais [41], uptake of apoptotic neutrophils by macrophages and dendritic cells (DCs) after *L. major* infection can limit the activation of phagocytes, leading to better parasite survival. This could explain the higher parasite load within ZCL compared to SCL lesions. Furthermore, as previously described in American CL and Ethiopian CL [27, 30], our results showed that the inflammatory infiltrate of ZCL and SCL lesions is primarily composed of T cells, followed by macrophages, very few B cells, and no NK cells. T cells play a critical role in the clinical presentation and course of leishmaniasis. In our case, a slight predominance of CD4^+^ T cells over CD8^+^ T cells was observed within lesions of both forms of CL studied. These results are consistent with those described for CL due to *L. braziliensis*, showing a statistically significant positive correlation between the intensity of the inflammatory infiltrate and the frequency of CD4^+^, CD8^+^ T cells, and CD68^+^ cells [16]. In addition, it was demonstrated that the healing process was associated with a decrease of CD4 and an increase of CD8, leading to similar CD4 and CD8 proportions [11, 15, 18]. These cells are expanded in long-term healed cutaneous leishmaniasis (hCL) patients [15]. Indeed, analysis of their functional characteristics, determined in *ex vivo* blood mononuclear cells, showed a reduction in the percentage of activated *Leishmania*-responder CD4^+^ and CD8^+^ T cells in hCL, associated with the time elapsed since clinical cure with evident expansion of effector memory T cells [32]. This is consistent with the results described by Zaph [44] showing that in a mice model, immunity to *L. major* is mediated by at least two distinct populations of CD4^+^ T cells: short-lived pathogen-dependent effector cells and long-lived pathogen-independent central memory cells.

T cells operate through production of cytokines and chemokines in shaping the nature of the immune response to *Leishmania* infection in both human and experimental models of the disease [2, 14, 42]. Herein, using RT-qPCR, we showed that IFN-γ and IL-10 mRNA were detected within most lesion samples tested. The IL-10 mRNA levels were comparable within both lesions, whereas IFN-γ mRNA levels were higher within SCL lesions. A positive correlation was found between mRNA levels of both cytokines within ZCL lesions, confirming our previous results [23], whereas no correlation was detected within SCL lesions. Interestingly, the ratio of IFN-γ/IL-10 levels indicated a predominance of Th1 response, which may explain the low levels of IL-13 mRNA detected within some of studied lesions. This result is consistent with findings previously described in CL caused by *L. braziliensis* or *L. major*, showing a predominance of Th1 cytokines (IL-2 and IFN-γ) over Th2 cytokines (IL-4) [12, 23, 26, 33]. An association between the mRNA levels of IL-10 and IFN-γ and lesion age and surface (size), as well as the parasite load, was also evaluated. In SCL lesions, IL-10 mRNA levels tend to decrease depending on lesion age, and conversely it increased as a function of the parasite number. A similar result was also observed for the ZCL lesion. Together, these results suggest that IL-10 is produced at the early stage of the development of lesions. This is consistent with data described in LCL lesions due to *L. guyanensis*, in which a Th2 response (IL-4 and/or IL-13) transiently predominates during the early phase of infection, followed by the development of a Th1 (IFN-γ) response during the late course of lesion development [9]. Authors have suggested that healing of lesions requires a T-cell-mediated immune response with IFN-γ production that can enhance the development of Th1 immune response and induce leishmanicidal activation of macrophages. In contrast, this is not found within ZCL lesions, in which the IFN-γ mRNA expression levels tend to decrease depending on lesion age. In our study, most biopsies were taken from late lesions (lesion age ranging from 30 to 360 days), which might explain the discrepancies with results described by Bourreau and colleagues who used biopsies from lesions aging less than 60 days. Furthermore, in SCL lesions, IFN-γ mRNA levels decreased with greater lesion size. This result is contradictory with that described by Antonelli and colleagues showing that inflammatory cytokines (IFN-γ and TNF-α), produced by PBMCs stimulated *ex vivo* with soluble *Leishmania* antigen (SLA), were associated with greater lesions size, suggesting their importance in the induction of tissue damage [1]. Similarly, in tegumentary leishmaniasis caused by *L. braziliensis*, there is evidence that increased production of IFN-γ, TNF-α and absence of IL-10 is associated with a strong inflammatory reaction and development of cutaneous and mucosal ulcers [17]. This discrepancy may be attributed to the difference in the age of lesions included in each study. Indeed, in our study SCL lesion age is higher than 60 days; however in the study done by Faria and colleagues, the ages of the active lesions ranged from 30 to 45 days for both CL and ML lesions. Nevertheless, these discrepancies could be attributed to the *Leishmania* species causing the disease.

Thereby, it is clear that the ability of the host to mount a cell-mediated immune response against the parasite is crucial for the defense against *Leishmania*. The composition of the cell populations recruited in the early phase of the infection seems to be essential for infection outcomes. Thus, we assessed the expression of pro-inflammatory chemokines, interleukin-8 (IL-8), and monocyte chemotactic factor-1 (MCP-1) within ZCL and SCL lesions. IL-8 mRNA was detected at higher levels within ZCL lesions compared to SCL lesions. IL-8 is one of the earliest and most abundant chemokines produced during acute inflammatory responses [24]. It is mainly responsible for neutrophil chemotaxis, which could explain the high number of polynuclears detected within ZCL lesions compared to SCL lesions. Moreover, this study reported expression of MCP-1 mRNA at similar levels within ZCL and SCL lesions. CCL2/MCP-1 induces the early recruitment of inflammatory monocytes and the leishmanicidal activities in murine monocytes [19]. This effect is enhanced by IFN-γ and abrogated by IL-4 [36]. However, no association was found with age nor with the size of lesions. These results are contradictory to those described by Ritter and colleagues showing high expression of CCL2/MCP-1, CXCL9/MIG, CXCL10/ IP-10 and only low amounts of CCL3/MIP-1α within lesions of localized cutaneous leishmaniasis (LCL) due to *L. mexicana*, while lesions of chronic diffuse cutaneous leishmaniasis (DCL) are dominated by the expression of CCL3/MIP-1 alpha, suggesting the implication of CCL2/MCP-1 in the healing process [35, 37]. MCP-1 expression described in *L. mexicana* active LCL lesions was found in the delayed type hypersensitivity (DTH) skin response to *Leishmania* antigen in subjects with healed lesions or subclinical infection [43].

Altogether, our results showed a clinical and histological difference between ZCL and SCL lesions due to *L. major* and *L. infantum*, respectively. Current data showed a predominance of Th1 within both lesions. Comparable levels of expression of IL-10 and MCP-1 mRNA were found within both CL lesions. However, a significant difference was found on expression of IFN-γ and IL-8 mRNA. ZCL lesions were characterized by high levels of IL-8 associated with a large number of polynuclears within the dermal infiltrate. More *in vivo* studies are needed to determine the involvement of these cells in *L. major* infection. However, higher levels of IFN-γ were detected within SCL lesions and such levels decreased according to lesion size. It is not excluded that this discrepancy might be attributed to the difference in the age of ZCL and SCL lesions enrolled in the present study, hence the importance of including groups matched for lesion age. Most of the studies on CL due to *L. braziliensis* or *L. mexicana* suggest that differential gene expression of cytokines and chemokines found in skin lesions from CL patients is associated with the time course of lesions [9, 13, 17, 37]. For Old World CL due to *L. major* and *L. infantum*, further analysis including biopsies from early lesions would be needed to track down the role of theses cytokines and chemokines in the development or healing of the infection.

## References

[1] Antonelli LR, Dutra WO, Almeida RP, Bacellar O, Carvalho EM, Gollob KJ. 2005 Activated inflammatory T cells correlate with lesion size in human cutaneous leishmaniasis. Immunology Letters, 101(2), 226–230. 16083969 10.1016/j.imlet.2005.06.004

[2] Antoniazi S, Price HP, Kropf P, Freudenberg MA, Galanos C, Smith DF, Müller I. 2004 Chemokine gene expression in toll-like receptor-competent and -deficient mice infected with *Leishmania major*. Infection and Immunity, 72, 5168–5174. 15322011 10.1128/IAI.72.9.5168-5174.2004 PMC517484

[3] Aoun K, Bouratbine A, Harrat Z, Guizani I, Mokni M, Bel Hadj Ali S, Ben Osman A, Belkaïd M, Dellagi K, Ben Ismaïl R. 2000 Données épidémiologiques et parasitologiques concernant la leishmaniose cutanée sporadique du nord tunisien. Bulletin de la Société de Pathologie Exotique, 93(2), 101–103. 10863611

[4] Aoun K, Bouratbine A. 2014 Cutaneous leishmaniasis in North Africa: a review. Parasite, 21, 14. 24626301 10.1051/parasite/2014014 PMC3952656

[5] BenSaid M, Guerbouj S, Saghrouni F, Fathallah-Mili A, Guizani I. 2006 Occurrence of *Leishmania* infantum cutaneous leishmaniasis in central Tunisia. Transaction of the Royal Society of Tropical Medicine and Hygiene, 100(6), 521–526. 10.1016/j.trstmh.2005.08.012 16356518

[6] Bettaieb J, Toumi A, Chlif S, Chelghaf B, Boukthir A, Gharbi A, Ben Salah A. 2014 Prevalence and determinants of *Leishmania major* infection in emerging and old foci in Tunisia. Parasites & Vectors, 7(1), 386. 25142220 10.1186/1756-3305-7-386 PMC4262385

[7] Bouratbine A, Aoun K, Ghrab J, Harrat Z, Ezzedini MS, Etlijani S. 2005 Spread of *Leishmania killicki* to Central and South West Tunisia. Parasite, 12(1), 59–63. 15828583 10.1051/parasite/2005121059

[8] Bourreau E, Prévot G, Gardon J, Pradinaud R, Launois P. 2001 High intralesional interleukin-10 messenger RNA expression in localized cutaneous leishmaniasis is associated with unresponsiveness to treatment. Journal of Infectious Disease, 184(12), 1628–1630. 10.1086/324665 11740743

[9] Bourreau E, Pascalis H, Prévot G, Kariminia A, Jolly N, Milon G, Buffet P, Michel R, Meynard JB, Boutin JP, Aschimoff D, Launois P. 2003 Th2 responses predominate during the early phases of infection in patients with localized cutaneous leishmaniasis and precede the development of Th1 responses. Infection and Immunity, 71(4), 2244–2246. 12654849 10.1128/IAI.71.4.2244-2246.2003 PMC152072

[10] Bousslimi N, Aoun K, Ben-Abda I, Ben-Alaya-Bouafif N, Raouane M, Bouratbine A. 2010 Epidemiologic and clinical features of cutaneous leishmaniasis in southeastern Tunisia. American Journal of Tropical Medicine and Hygiene, 83(5), 1034–1039. 21036833 10.4269/ajtmh.2010.10-0234 PMC2963965

[11] Brodskyn CI, Barral A, Boaventura V, Carvalho E, Barral-Nett M. 1997 Parasite-driven in vitro human lymphocyte cytotoxicity against autologous infected macrophages from mucosal leishmaniasis. Journal of Immunology, 159, 4467–4473. 9379046

[12] Cáceres-Dittmar G, Tapia FJ, Sánchez MA, Yamamura M, Uyemura K, Modlin RL, Bloom BR, Convit J. 1993 Determination of the cytokine profile in American cutaneous leishmaniasis using the polymerase chain reaction. Clinical Experimental Immunology, 91(3), 500–505. 8443970 10.1111/j.1365-2249.1993.tb05931.x PMC1554703

[13] Costa-Silva MF, Gomes LI, Martins-Filho OA, Rodrigues-Silva R, Freire Jde M, Quaresma PF, Pascoal-Xavier MA, Mendes TA, Serakides R, Zauli DA, Campi-Azevedo AC, Melo MN, Gontijo CM, Peruhype-Magalhães V, Teixeira-Carvalho A. 2014 Gene expression profile of cytokines and chemokines in skin lesions from Brazilian Indians with localized cutaneous leishmaniasis. Molecular Immunology, 57(2), 74–85. 24084096 10.1016/j.molimm.2013.08.008

[14] Cummings HE, Tuladhar R, Satoskar AR. 2010 Cytokines and their STATs in cutaneous and visceral leishmaniasis. Journal of Biomedicine and Biotechnology, 2010, 294389. 20300429 10.1155/2010/294389 PMC2840379

[15] Da-Cruz AM, Bittar R, Mattos M, Oliveira-Neto MP, Nogueira R, Pinho-Ribeiro V, Azeredo-Coutinho RB, Coutinho SG. 2002 T-cell-mediated immune responses in patients with cutaneous or mucosal leishmaniasis: long-term evaluation after therapy. Clinical and Diagnostic Laboratory Immunology, 9(2), 251–256. 11874860 10.1128/CDLI.9.2.251-256.2002 PMC119941

[16] Dutra WO, Faria DR, Lima Machado PR, Guimarães LH, Schriefer A, Carvalho E, Gollob KJ. 2011 Immunoregulatory and effector activities in human cutaneous and mucosal leishmaniasis: understanding mechanisms of pathology. Drug Development Research, 72(6), 430–436. 23504276 10.1002/ddr.20449 PMC3595558

[17] Faria DR, Gollob KJ, Barbosa J, Jr., Schriefer A, Machado PR, Lessa H, Carvalho LP, Romano-Silva MA, de Jesus AR, Carvalho EM, Dutra WO. 2005 Decreased in situ expression of interleukin-10 receptor is correlated with the exacerbated inflammatory and cytotoxic responses observed in mucosal leishmaniasis. Infection and Immunity, 73(12), 7853–7859. 16299275 10.1128/IAI.73.12.7853-7859.2005 PMC1307048

[18] Faria DR, Souza PE, Durães FV, Carvalho EM, Gollob KJ, Machado PR, Dutra WO. 2009 Recruitment of CD8 T cells expressing granzyme A is associated with lesion progression in human cutaneous leishmaniasis. Parasite Immunology, 31, 432–439. 19646207 10.1111/j.1365-3024.2009.01125.x PMC2764276

[19] Goncalves R, Zhang X, Cohen H, Debrabant A, Mosser DM. 2011 Platelet activation attracts a subpopulation of effector monocytes to sites of *Leishmania major* infection. Journal of Experimental Medicine, 208, 1253–1265. 21606505 10.1084/jem.20101751 PMC3173254

[20] Gollob KJ, Viana AG, Dutra WO. 2014 Immunoregulation in human American leishmaniasis: balancing pathology and protection. Parasite Immunology, 36(8), 367–376. 24471648 10.1111/pim.12100 PMC4113557

[21] Haouas N, Gorcii M, Chargui N, Aoun K, Bouratbine A, Messaadi Akrout F, Masmoudi A, Zili J, Ben Said M, Pratlong F, Dedet JP, Mezhoud H, Azaiez R, Babba H. 2007 Leishmaniasis in central and southern Tunisia: current geographical distribution of zymodemes. Parasite, 14(3), 239–246. 17933302 10.1051/parasite/2007143239

[22] Jardim A, Funk V, Caprioli RM, Olafson RW. 1995 Isolation and structural characterisation of the *Leishmania donovani* kinetoplastid membrane protein-11, a major immunoreactive membrane glycoprotein. Biochemistry Journal, 305, 307–313. 10.1042/bj3050307 PMC1136464 7826346

[23] Louzir H, Melby PC, Ben Salah A, Marrakchi H, Aoun K, Ben Ismail R, Dellagi K. 1998 Immunologic determinants of disease evolution in localized cutaneous leishmaniasis due to *Leishmania major*. Journal of Infectious Disease, 177, 1687–1695. 10.1086/515297 9607850

[24] Luster AD. 1998 Chemokines: chemotactic cytokines that mediate inflammation. New England Journal of Medicine, 338, 436–445. 9459648 10.1056/NEJM199802123380706

[25] Martins AL, Barreto JA, Lauris JR, Martins AC. 2014 American tegumentary leishmaniasis: correlations among immunological, histopathological and clinical parameters. Anais Brasileiros De Dermatologia, 89(1), 52–58. 24626648 10.1590/abd1806-4841.20142226 PMC3938354

[26] Melby PC, Andrade-Narvaez FJ, Darnell BJ, Valencia-Pacheco G, Tryon VV, Palomo-Cetina A. 1994 Increased expression of proinflammatory cytokines in chronic lesions of human cutaneous leishmaniasis. Infection and Immunity, 62(3), 837–842. 8112853 10.1128/iai.62.3.837-842.1994 PMC186190

[27] Modlin RL, Tapia FJ, Bloom BR, Gallinoto ME, Castes M, Rondon AJ, Rea TH, Convit J. 1985 In situ characterization of the cellular immune response in American cutaneous leishmaniasis. Clinical and Experimental Immunology, 60(2), 241–248. 3159527 PMC1577040

[28] Mokni M, Mebazaa A, Boubaker S. 2011 Histology of cutaneous leishmaniasis. Annales de Dermatologie et de Vénéréologie, 138(4), 354–356. 21497266 10.1016/j.annder.2010.10.018

[29] Nicolas L, Prina E, Lang T, Milon G. 2002 Real-time PCR for detection and quantification of *Leishmania* in mouse tissues. Journal of Clinical Microbiology, 40, 1666–1669. 11980939 10.1128/JCM.40.5.1666-1669.2002 PMC130941

[30] Nilsen R, Mshana RN. 1987 In situ characterization of the cutaneous immune response in Ethiopian cutaneous leishmaniasis. Scandinavian Journal of Immunology, 26(5), 503–512. 3120304 10.1111/j.1365-3083.1987.tb02284.x

[31] Nylén S, Eidsmo L. 2012 Tissue damage and immunity in cutaneous leishmaniasis. Parasite Immunology, 34(12), 551–561. 23009296 10.1111/pim.12007

[32] Pereira-Carvalho R, Mendes-Aguiar CO, Oliveira-Neto MP, Covas CJ, Bertho AL, Da-Cruz AM, Gomes-Silva A. 2013 *Leishmania braziliensis*-reactive T cells are down-regulated in long-term cured cutaneous leishmaniasis, but the renewal capacity of T effector memory compartments is preserved. PLoS One, 8(11), e81529. 24303052 10.1371/journal.pone.0081529 PMC3841203

[33] Pirmez C, Yamamura M, Uyemura K, Paes-Oliveira M, Conceição-Silva F, Modlin RL. 1993 Cytokine patterns in the pathogenesis of human leishmaniasis. Journal of Clinical Investigation, 91(4), 1390–1395. 8473490 10.1172/JCI116341 PMC288111

[34] Reithinger R, Dujardin JC, Louzir H, Pirmez C, Alexander B, Brooker S. 2007 Cutaneous leishmaniasis. Lancet Infectious Diseases, 7(9), 581–596. 17714672 10.1016/S1473-3099(07)70209-8

[35] Ritter U, Moll H, Laskay T, Brocker E, Velazco O, Becker I, Gillitzer R. 1996 Differential expression of chemokines in patients with localized and diffuse cutaneous American leishmaniasis. Journal of Infectious Diseases, 173, 699–709. 8627035 10.1093/infdis/173.3.699

[36] Ritter U, Moll H. 2000 Monocyte chemotactic protein-1 stimulates the killing of Leishmania major by human monocytes, acts synergistically with IFN-γ and is antagonized by IL-4. European Journal of Immunology, 30, 3111–3120. 11093125 10.1002/1521-4141(200011)30:11<3111::AID-IMMU3111>3.0.CO;2-O

[37] Ritter U, Körner H. 2002 Divergent expression of inflammatory dermal chemokines in cutaneous leishmaniasis. Parasite Immunology, 24(6), 295–301. 12102714 10.1046/j.1365-3024.2002.00467.x

[38] Russo J, Sheriff F, Lopez de Cicco R, Pogash TJ, Nguyen T, Russo IH. 2014 Methodology for studying the compartments of the human breast, in Techniques and Methodological Approaches in Breast Cancer Research, Russo J, Russo IH, Editors. Erwei Song, Hai Hu p. 75–102.

[39] Salah AB, Kamarianakis Y, Chlif S, Alaya NB, Prastacos P. 2007 Zoonotic cutaneous leishmaniasis in central Tunisia: spatio temporal dynamics. International Journal of Epidemiology, 36(5), 991–1000. 17591639 10.1093/ije/dym125

[40] Scott P. 2005 Immunologic memory in cutaneous leishmaniasis. Cellular Microbiology, 7(12), 1707–1713. 16309457 10.1111/j.1462-5822.2005.00626.x

[41] Scott P, Novais FO. 2016 Cutaneous leishmaniasis: immune responses in protection and pathogenesis. Nature Reviews Immunology, 16(9), 581–592. 10.1038/nri.2016.72 27424773

[42] Teixeira MJ, Teixeira CR, Andrade BB, Barral-Netto M, Barral A. 2006 Chemokines in host-parasite interactions in leishmaniasis. Trends in Parasitology, 22, 32–40. 16310413 10.1016/j.pt.2005.11.010

[43] Valencia-Pacheco G, Loría-Cervera EN, Sosa-Bibiano EI, Canché-Pool EB, Vargas-Gonzalez A, Melby PC, Andrade-Narvaez FJ. 2014 In situ cytokines (IL-4, IL-10, IL-12, IFN-γ) and chemokines (MCP-1, MIP-1α) gene expression in human *Leishmania* (*Leishmania*) *mexicana* infection. cytokine, 69(1), 56–61. 25022962 10.1016/j.cyto.2014.05.016

[44] Zaph C, Uzonna J, Beverley SM, Scott P. 2004 Central memory T cells mediate long-term immunity to *Leishmania major* in the absence of persistent parasites. Nature Medicine, 10(10), 1104–1110. 10.1038/nm1108 15448686

